# Adherence to prophylaxis in adolescents and young adults with severe haemophilia: a qualitative study with healthcare professionals

**DOI:** 10.1080/21642850.2020.1718501

**Published:** 2020-01-28

**Authors:** S. van Os, N. Ryder, D. P. Hart, N. Troop

**Affiliations:** a Psychology and Sport Sciences Department, School of Life and Medical Sciences, University of Hertfordshire, Hatfield, UK; b The Royal London Hospital Haemophilia Centre, Barts Health NHS Trust, London, UK; c Barts and The London School of Medicine and Dentistry, QMUL, London, UK

**Keywords:** Adherence, haemophilia, prophylaxis, adolescents and young adults, personalised treatment

## Abstract

**Aim:** to examine healthcare professionals’ (HP) perceptions and experiences in relation to adherence to prophylactic treatment among young people living with haemophilia (YPH).

**Methods:** All HPs in four haemophilia centres across England and Wales were invited to participate, and all HPs who agreed to take part (*n* = 6) were interviewed. Interviews were audio-recorded, transcribed and then analysed using Interpretative Phenomenological Analysis (IPA).

**Results:** HPs estimate that generally young people with haemophilia keep to their treatment regimen well, although they also recognise that adherence may fluctuate with many patients going through shorter periods of non-adherence. The increasingly personalised or flexible approach to prophylaxis makes it harder to assess adherence. The main themes identified through IPA included (1) HPs’ suggest that adherence fluctuates (2) Non-adherence is mainly driven by lifestyle and developmental, social and family factors, and (3) Education, HPs’ sensitivity to individual needs, and psychological and peer support are key facilitators of good adherence.

**Conclusion:** The increasingly flexible approach to prophylaxis requires a new way of thinking about, and assessment of, adherence. More personalised treatment regimen can be more complicated and may, therefore, lead to accidental non-adherence. The results of this study with HPs complement those of a previous qualitative study with patients but place greater emphasis on a broader perspective on understanding drivers of non-adherence as well as understanding strategies to improve adherence in the minority of patients who appear to struggle.

## Introduction

Haemophilia is an inherited bleeding disorder that is caused by a deficiency in one of the coagulation (blood clotting) factors. Haemophilia A is caused by a deficiency in factor VIII and Haemophilia B is caused by a deficiency in factor IX. Haemophilia occurs mostly in males, and there are approximately 9400 people with either Haemophilia A or B in the U.K. (United Kingdom Haemophilia Centre Doctors’ Organisation (UKHCDO) Annual Report, 2015/2016).

Most young people living with severe haemophilia in the U.K. follow a preventative treatment regimen (known as prophylaxis), which consists of several intravenous injections of factor concentrate. Prophylaxis increases the concentration of factor in the blood, which reduces the risk of joint bleeds and the resulting joint damage (Fischer, 2012; Manco-Johnson et al., 2007), and intracranial and muscle bleeds (Ljung, 2009; Witmer et al., 2011), whilst also improving quality of life (Richards et al., 2010).

It is crucial that patients keep to their prophylactic treatment regimen, as even low levels of non-adherence can cause significant bleeds (Collins et al., 2009; Hacker, Geraghty, & Manco-Johnson, 2001; Manco-Johnson et al., 2007), and may result in joint damage leading to poorer physical and emotional wellbeing (Manco-Johnson et al., 2007; du Treil, Rice, & Leissinger, 2007).

Traditional weight-based prophylaxis regimens are becoming less common as many patients follow more individualised treatment plans designed around their lifestyle, planned activities, individual bleeding pattern, condition of their musculoskeletal system, and measurement of coagulation factor in their blood (Collins, 2012; Fischer, 2012; Makris, 2012; van Os, Troop, Ryder, & Hart, 2018). The findings from a recent study (van Os, Troop, Sullivan, & Hart, 2017) with a large U.K. sample of young people with haemophilia (YPH) on prophylaxis suggest that adherence was good with most patients keeping to their agreed treatment regimen most of the time (just 18% had a score that indicated that they were non-adherent a significant proportion of the time). However, it is important to note that measures that assess adherence in haemophilia (such as the Duncan, Kronenberger, Roberson, & Shapiro, 2010), are based on traditional approaches to treatment, limiting their utility in the context of increasingly personalised treatment. A qualitative study carried out as part of the same research programme, which investigated the lived experiences of YPH (van Os et al., 2018) found that the increasingly flexible and personalised approach has implications for adherence. This is because it may lead to confusion around treatment frequency and dosing, which in turn could lead to accidental non-adherence, which is distinct from both skipping and forgetting. The current study, also part of the same research programme, adds the perspective of haemophilia healthcare professionals (HP). This study aims to investigate HPs’ experiences and perceptions in relation to adherence to prophylaxis, as well as other self-management behaviours such as treating bleeds, attending clinic appointments, completing treatment logs, and avoiding activities that are considered too risky for people with severe haemophilia (e.g. contact sports such as rugby and football).

## Materials and methods

### Interpretative Phenomenological Analysis (IPA)

IPA is a qualitative research approach that aims to offer insights into lived experience and how participants themselves make sense of their experiences (Smith, 2010). IPA is concerned with exploring personal perceptions or accounts of events or states, rather than trying to produce objective statements of experiences. However, IPA recognises that access to the individual participant's world is dependent on the researcher making sense of that other personal world through a process of interpretation. IPA is also strongly idiographic in its approach, with an emphasis on detailed analysis of each unique personal experience before moving to detailed analysis of other cases.

To ensure that standards in relation to rigour, validity and credibility were met, Yardley’s (2000, 2008) ‘*Characteristics of good qualitative research’* were used as the guiding principles. During the research process there was a particular focus on the transparency of data presentation, reflexivity around the researcher's own assumptions, and consideration of alternative perspectives.

### Recruitment

The number of participants in IPA studies is usually small, to enable a detailed case-by-case analysis that gives full appreciation to each individual participant's account (Collins & Nicholson, 2002; Smith & Osborn, 2003). IPA studies employ a fairly homogenous sample, allowing comparison of similarities and differences within a group that has been defined as similar in relation to the phenomenon being investigated (Reid, Flowers, & Larkin, 2005). Therefore inclusion criteria for participants in this study were very specific, participants had to be HPs involved in the care for YPH (12–25 years) with severe haemophilia who follow a prophylactic treatment regimen. All HPs who were specifically involved in the care of YPH in four haemophilia centres across England and Wales (approximately 5 doctors and 9 nurses) were invited to participate in this study via email, telephone and in person. Eligible participants included physiotherapists, doctors (Registrar, Consultant, Senior Consultant and Centre Director) and nurses (across all levels of seniority). All participants who agreed to take part, and who were available (*n* = 6) were interviewed. Unfortunately no physiotherapists were available to take part in the study during the data collection period.

Potential participants were sent an information sheet about the study via email and encouraged to email the researcher if they were interested in taking part. Potential participants who did not respond within one month were contacted again via email or telephone. The researcher then contacted all HPs who had indicated that they would like to participate to arrange the interview. Informed written consent was obtained on the day of the interview.

### Participants

Participants were 4 haemophilia nurses and 2 haematologists. Please see Table 1 for details.

**Table 1.** Participant characteristics.

Participant code	Role	Sex
HP 1	Nurse	Female
HP 2	Nurse	Female
HP3	Nurse	male
HP4	Haematologist	Female
HP5	Haematologist	male
HP6	Nurse	Female

### Data collection

IPA studies require data collection methods that will result in rich, detailed and first-person accounts of the experiences and or phenomena under investigation. Semi-structured interviews are considered a good data collection method for IPA studies, as they facilitate a more informal and free-flow interview, which enables the researcher to follow cues from participants and probe areas of interest that appear particularly relevant to each participant's experiences (Brocki & Wearden, 2006).

A semi-structured discussion guide was developed based on literature in relation to treatment adherence among young people diagnosed with chronic health conditions (Alvin, Rey, & Frappier, 1995; Arias Llorente, Bousoño García, & Díaz Martín, 2008; Iannotti et al., 2006; La Greca et al., 1995; La Greca & Bearman, 2002; Salema, Elliott, & Glazebrook, 2011; Shaw, 2001), HP involvement in relation to adherence (DiMatteo et al., 1993; Street, Makoul, Arora, & Epstein, 2009), haemophilia (De Moerloose, Urbancik, van den Berg, & Richards, 2008; Duncan et al., 2010; Khair, 2013; Llewellyn, Miners, Lee, Harrington, & Weinman, 2003; Thornburg, 2008; du Treil et al., 2007), and guidance from Smith and Osborn (2008). Feedback collected from focus groups with patients, parents, and HPs was used to fine-tune the discussion guide. All interviews were undertaken by the same interviewer as part of their Ph.D. project, and took place in private rooms in the haemophilia centres. Participants were not on duty and were, therefore, able to focus their attention solely on the interview. Questions were delivered in a non-directive and open style to encourage participants to share their experiences in their own words. Participants were reassured that information given would be confidential.

At the start of the interview, participants were invited to talk about their experience of working in the haemophilia centre (e.g. ‘Tell me about what an average day is like for you in the treatment centre’). To gain further insights HPs were invited to describe their experiences and perceptions in relation to prophylaxis, and adherence to this treatment by adolescent and young adult patients (‘How well do your patients adhere to their prophylaxis?’ ‘Tell me about your experiences with non-adherent patients’. ‘In your experience what kinds of things might interfere with adherence?’ etc.). Lastly, HPs were encouraged to talk about potential (healthcare) improvements they felt may help them and/or their patients to improve adherence (‘What would help you to improve adherence?’ etc.). The order in which the above subjects were discussed was flexible, and driven by participants themselves. In most of the interview, the HPs volunteered much of the information they shared without being prompted or asked specific questions. Interviews lasted between 35 and 95 minutes and were audio-recorded, transcribed and analysed following IPA principles and guidelines (Smith, 2003; Smith & Osborn, 2008).

### Data analysis

During the analytical process in IPA studies, the researcher engages in a double hermeneutic (Smith, Flowers, & Larkin, 2009), to try and make sense of the participant attempting to make sense of their experiences.

The transcription of the interview recordings is often seen as the first phase of analysis in IPA studies. Firstly an ‘everything audible’ version was produced to ensure that future transcriptions stemmed from the maximum possible transcribed content. After removing all irrelevant noises and identifying information, validation was carried out by an independent researcher who read transcripts while listening to the recordings. The main researcher then re-read the transcripts to ensure they were ready for analysis.

As suggested by Smith and Osborn (2003) IPA was conducted on a single interview in its entirety first before the remainder of the transcripts were taken through the analytical process. The analysis started by making comments and annotations (in Nvivo) while reading and re-reading the transcript. After detailed review, the coding was then refined, and the Nvivo coded transcript was then exported into an Excel spreadsheet. This spreadsheet contained one line for each code, and columns providing more information about the code (e.g. code name, example quote, notes/reflections, and themes to which the code may belong). The themes were then reviewed with the aim to identify overarching themes, which were reported in an additional column. The researcher continually returned to the transcripts throughout this process, to ensure that the themes and superordinate themes were closely connected to what participants had actually said during the interviews. Finally, the overarching themes were refined and presented in a table together with each of their subordinate themes. An independent researcher with no prior knowledge of haemophilia or treatment adherence then reviewed these themes, and their relation to the initial transcript and each other. This process highlighted certain areas in the analysis that required attention to ensure that the reporting of the results would be clear and transparent.

### Ethics statement

Ethical approval was obtained from the NHS National Research Ethics Service Committee, London – City Road & Hampstead (NRES, Ref: 12/LO/2030). This approval covers both providers and patients.

## Results

### Superordinate and subordinate themes

Here we present three superordinate themes in relation to HPs perceptions and experiences of adherence to prophylaxis among their adolescent and young adult patients. Table 2 presents the superordinate themes with their related subordinate themes, and for which participants each of the themes was relevant.

**Table 2.** Superordinate and subordinate themes for each participant.

Superordinate themes	Subordinate themes	Participants					
		1	2	3	4	5	6
Healthcare professionals suggest that adherence fluctuates	Adherence fluctuates	x		x	x	x	x
	Timing of injections	x		x	x	x	x
	Variability of symptoms (bleeds)	x		x	x	x	x
	Haemtrack and adherence	x		x	x	x	x
Non-adherence is mainly driven by lifestyle and developmental, social and family factors	Lifestyle and time management	x	x	x	x	x	x
	Venepuncture issues		x	x	x		x
	Not wanting to be different	x	x		x	x	x
	Absence of symptoms			x	x	x	x
	Family and social issues		x		x	x	x
Education, HPs’ sensitivity to individual needs, and psychological and peer support are key facilitators for good adherence	Education	x	x	x	x	x	x
	Psychological support	x	x	x	x	x	x
	Peer support	x	x	x	x	x	x
	Being sensitive to individual needs	x	x	x	x	x	x
	Regular contact and continuity of staff	x		x	x	x	x
	Collaboration with schools and community	x		x		x	x

### Theme 1: healthcare professionals suggest that adherence fluctuates

HPs commented on adherence mostly in terms of patients keeping to their agreed treatment regimen, but also in relation to other self-care behaviours such as treating bleeds, logging treatments and attending clinical appointments. Their patients’ adherence, particularly in relation to prophylaxis, appears to be one of their key concerns. HPs suggested that they focus a significant proportion of their working week on adherence, e.g. by following up individual patients, checking Haemtrack treatment logs, and having face-to-face meetings with patients. Although adherence is a concern across the patient population, HPs are particularly concerned about younger patients who are in the pre-adolescent and adolescent period, as adherence appears a particular problem during this developmental stage.

Although HPs found it difficult to estimate adherence to treatment in terms of percentages, they agreed that adherence is generally good for the majority of their patients. Good in this context means that patients keep to their treatment regimen most of the time, dramatically reducing the risk of bleeding.

Gosh, quite hard to think of [laughs] I don't know if I could put a percentage. But I would say that the vast majority are actually quite adherent. I’d say the vast – I don't know, it's kind of [pause] 75%. I don't know, it's kind of a random number, but I’d say – or even maybe more. I think, the bulk are more or less fairly adherent. (HP1)

However, HPs explained that a minority of patients struggle with their treatment and that even patients who tend to keep to their treatment regimen can go through periods of non-adherence. HPs, in particular nurses, explained that they spend a lot of their time and effort on this relatively small group of non-adherent patients, either to work with them to improve their adherence or to treat them for bleeds that are the result of their non-adherence. Having to concentrate on these non-adherent patients can be difficult for the haemophilia team, as they often feel their efforts do not necessarily result in better adherence or fewer bleeds. This sometimes leaves HPs feeling frustrated and worried about patients who continue to be non-adherent, but also concerned that they do not have enough time to look after their other patients.

Three HPs estimated adherence among YPH in terms of percentages. They estimated that although nearly all (90–100%) of their patients take their prophylaxis, significantly fewer patients (50%; 70% and 80% respectively) take it at the agreed time. They suggest that most non-adherence is about the timing of treatment, with patients taking their prophylaxis in the afternoon or evening instead of the morning. Patients who do not take their treatment in the morning may not be adequately protected during the day, when they are most likely to be at risk of bleeding. HPs speak to patients about the importance of timing regularly, and try to work with patients to find a way to fit treatment in. However, it appears that some patients are simply not able or willing to consider taking their prophylaxis before they leave the house in the morning. This can leave HPs feel powerless, as they feel there is nothing they can do to get these patients back on track with their treatment.

HPs explained that in recent years patients have increasingly been encouraged to tailor their treatment around their activities and lifestyle, meaning that treatment plans have become less regimented. Particularly patients who live an active lifestyle may change their treatment according to their physical activities, to ensure they are protected at the times that they are at most risk. It is therefore not always straightforward to ascertain or define whether someone is adherent. In some cases, one may argue that someone who follows a rigid three times per week regimen is non-adherent because they do not tailor their treatment to provide cover for activities.

So even though they are adhering to their regimen, actually they are sort of non-adherent because they are not managing around their activity. Or treating at the wrong time. (HP3)

HPs estimated that adherence to the other self-management behaviours is much lower and more likely to fluctuate over time. They felt that most patients will usually prioritise taking their prophylaxis because they recognise how important it is, but that completing the treatment log and attending clinic appointments are not usually prioritised in the same way. HPs expressed concern about patients who do not treat bleeds appropriately, as these bleeds often last longer and cause more damage. They felt that patients who present late with bleeds, or do not treat bleeds adequately, are often also those who struggle to keep to their treatment regimen, are less likely to log treatments on Haemtrack, or attend clinic appointments.

When talking about their experiences and perceptions in relation to adherence HPs clearly felt that they need to follow a more pragmatic approach, and accept that not all YPH will be able to follow their recommended regimen at all times. HPs tend to work with patients and/or families to find compromises that balance a reduction in treatment burden with making sure that the patient is still protected, particularly during times that they are at higher risk of bleeding.

So although there's lots of positives of daily treatment and that would be ideal in terms of levels. The practicality of that for some families is that it's not going to work. I suppose the compromise then is alternate days or we say “Monday, Wednesday and Friday and once at the weekend”. If that's what's prescribed, I would say 90–100% of people are doing that. I would say particularly in children that less than 50% of them would be doing it in the morning. I think it's after school, in the evening. I suppose it should be like brushing your teeth that you shouldn't do something without it, but that's not how it works for the families. (HP6)

Not all HPs agreed that bleeds necessarily indicate non-adherence. Some felt that patients who suffer frequent bleeds and those who stop attending clinic appointments are more likely to be non-adherent. However, others pointed out that there are patients who keep to their regimen religiously but still suffer frequent bleeds because the dosage or frequency of their treatment is not sufficient or due to existing joint damage or other factors that may cause bleeding.

HPs explained that the majority of patients who follow a prophylactic regimen are required to log each treatment on Haemtrack, a national, online treatment log. Haemtrack allows haemophilia centres to monitor patients remotely, to check whether they are taking their treatment as agreed and whether they are experiencing any bleeds. HPs felt that information logged on Haemtrack can be a good conversation starter with patients, and can help to illustrate the link between missed treatments (non-adherence) and bleeds.

I think Haemtrack is quite good because it's quite nice, not to check up on people but because it starts the conversation and it makes them actually talk about it and you can say, “Okay, well why you are missing it, is there things we can do to help” (HP4)

One concern about Haemtrack that was highlighted by several HP is that it is 100% reliant on the accuracy of information entered by patients. For instance, patients who do not want to admit that a bleed was caused by an activity they should not have been doing, may log these bleeds as breakthrough (spontaneous) bleeds. One HP felt that Haemtrack could, therefore, be used by commissioners to prove that prophylaxis does not work, as patients should not be having spontaneous bleeds while on prophylaxis. Another concern highlighted was that many patients appear to complete the log periodically, typically once a week or month, rather than after each treatment. This means that the data is not as informative or useful, particularly as many patients do not appear to complete/change the time they took each treatment, making it impossible to link bleeds to non-adherence.

### Theme 2: non-adherence is mainly driven by lifestyle and developmental, social and family factors

HPs felt that for many patients adherence to prophylaxis is a question of time and lifestyle. They showed a strong appreciation of the difficulties their patients face in trying to keep to their prophylactic regime during a busy period, or while going through a significant life event or change. HPs agreed that one of the most challenging aspects of prophylaxis is the timing of the injections. Although for many patients it would be best to take treatment in the morning before leaving home, HPs agreed that this is simply not achievable for all their patients.

I think that that's also why it's really important that we visit families at home because that gives you a much better reality of what family life is like for them. Families maybe where there's single parents, there's multiple children, that in the mornings before school is a rubbish time. As the health professional and the expert we’re saying “This is what you should be doing” but the reality of that happening is really, really difficult in terms of practical. (HP6)

Other issues that were suggested to be potential barriers to adherence include needle phobia, problems with venous access, YPH not wanting to be different from their peers, social and family issues, and an absence of symptoms making it difficult to understand the importance of taking prophylaxis.

HPs felt that they have a good range of tools available to support patients while learning to do their own treatment (e.g. implantable venous access devices, anaesthetic creams, distraction techniques, support from play therapists, peer support, etc.). Thanks to these tools most patients learn to do their injections without developing needle phobia. However, several HPs felt quite strongly that the way parents deal with haemophilia and the treatment has a strong influence and that issues such as needle phobia are much more likely to occur if parents struggle with needles or are anxious about treatment.

Sometimes I think it's affected by how the parents react to it and how they’re feeling about it. We do have play specialists, distraction, and we have local anaesthetic creams. If all of those things are in place in the beginning, what you see then in the centre is that we have boys that are three or four that will come in and accept it and it all happens really smoothly. Now that didn't happen without some other things happening before it. But I think it's more perhaps the parents struggle with a bit of needle phobia. And is that needle phobia or the thought of doing something so invasive to your child (HP6)

HPs explained that for some patients it is very hard to find a vein to inject to. These venous access issues can, in turn, cause treatment-related stress and anxiety, which is likely to affect adherence as patients may skip treatments when they are feeling anxious or are struggling to find a vein.

HPs suggested that most adolescent patients go through a stage of suboptimal adherence, ranging from occasional skipping to complete disengagement with treatment and the haemophilia team. They felt that this is partially because many adolescent patients feel that their haemophilia makes them different from their peers, which can cause resentment or embarrassment about having to take treatment.

Don't like to be different, don't like to be seen different. Don't want to do it, had enough of it, don't want to stick needles in themselves, no one else to stick needles in them, feel well in themselves, a desire to conform with peers. (HP5)

Adolescence is also a time during which many patients take over responsibility for their own treatment with less or little involvement from their parents. HPs find it very challenging to engage with patients while they go through this period, as patients tend to rebel against the haemophilia team as well as their parents. They clearly felt frustrated that they do not always have the knowledge or the resources to support these patients through this challenging period and help them stay on track with their prophylaxis to prevent bleeds and the related issues.

HPs explained that thanks to prophylaxis many patients now manage to get to adolescence without having experienced any severe bleeds, which makes it difficult for them to understand why they need to take their prophylaxis. They felt that the experience of a first serious bleed often encourages these non-adherent young patients to start taking their prophylaxis again. However, they also explained that for some patients this only works for a short period after which they stop taking treatment again, with more bleeds as a result. This vicious circle is often frustrating and worrying for HPs who look after these patients, particularly as they often feel there is not much they can do.

The relationship between patients and their parents and siblings was put forward as another key factor that influences adherence. HPs spend a significant proportion of their time and resources on helping patients who struggle with their treatment due to social and family issues.

And we’ve got another family where we have to do supervised prophylaxis for different reasons ‘cos their lives are crazy chaotic and they have huge dogs, like really big dogs in the house and there's nowhere safe to actually … These dogs are quite aggressive so there's no way you’d want anyone using a needle near a child with all this going on. So they come to the unit and get to the prophylaxis. (HP4)

It is clear that HPs are sensitive to individual circumstances, and appreciate that looking after a child with haemophilia can be stressful for both parents and healthcare professionals. HPs felt it is not always clear to them whether haemophilia itself is the cause of parental stress or anxiety, or whether stress or anxiety caused by outside factors make it harder to manage haemophilia for parents.

### Theme 3: education, HPs’ sensitivity to individual needs, and psychological and peer support are key facilitators for good adherence

Factors that facilitate adherence among YPH, according to HPs, include sensitivity to individual needs of parents and patients, psychosocial support, peer support and support for parents. HPs felt that patients’ knowledge and understanding about haemophilia and how prophylaxis works are crucial, as patients who are more aware tend to be more adherent. They, therefore, felt that education to improve patient and parental knowledge and understanding may help to improve adherence. Several interviewees emphasised that they felt that it is important to involve patients from an early age so that they can learn to do their treatment themselves gradually before they start secondary school.

The norm would be persuasion I think and gradual involvement of the child, and we do make it quite clear to the boys when they start on prophylaxis, when they’re going through prophylaxis in primary school, that the aim is that by the time they go to secondary school they’ll be giving their own treatment […] we encourage boys to start helping the parents at home, mixing up treatment, cleaning the skin, take needles out after finishing treatment, you know doing everything other than venepuncture. And then school holidays we try and get them to come to the unit so I guess depending on how they’re doing but certainly from the age of nine they’d be coming to the unit to try and learn some venepuncture (HP5)

HPs agreed that starting early also ensures that patients are used to doing their own treatment before they hit adolescence, which is a period during which they find it generally harder to engage patients. HPs felt that it is important to recognise that they are not necessarily trained to educate or provide psychosocial/emotional support and often do not have sufficient time or resources to work with patients or families who are struggling. HPs talked about situations in which they found it challenging to ascertain the exact nature of the issues that were affecting their patient, and felt that they could have done with help from specialist support staff.

if they were in-house it maybe they would be able to build it as part of the relationship with the centre in a more holistic sort of way — but I think there is a lot of psychosocial bits that as nurses we try to do our best but it would be nice if we had somebody who was a professional who knew how to deal, especially with, because some of them have some pretty major issues that, that, you know, sometimes I think we feel a bit lost with how to really deal with properly and support (HP1)

HPs suggested that particularly support from a play therapist, psychologist, or social worker can be helpful when working with a patient or family who are struggling with prophylaxis. Play therapists tend to work with younger children to, for instance, remove some of the anxiety that is attached to their hospital visits and treatment. Social workers may work with families on some of the social or relational barriers that interfere with treatment, whereas a psychologist may tackle issues around needle phobia and emotional or mental health issues that could impact on adherence. Although each of the above specialists have their own area of expertise, in reality it is unlikely that haemophilia centres have access to a play specialist, social worker and psychologist. It is therefore likely that these specialists work with patients and families in flexible ways that ‘borrow’ techniques from the other specialisms if and when needed.

The way in which support from psychosocial specialists is accessed appears to differ from centre to centre. Two HPs explained that they have access to a play specialist that is based at their hospital and that their team includes at least one psychologist and/or social worker who can identify psychosocial issues as they arise and work with patients before problems escalate.

I think it helps me sometimes because they’re looking from the outside, I’m involved in the moment, they’re just kind of observing what's happening and then they can offer some different advice. We’ve done lots since we had psychology embedded in our service, so there's families with children that are new to haemophilia so a new diagnosis, we do some joint work (HP6)

These HPs also described how they themselves felt psychologically or emotionally supported by the psychologist or social worker, helping them to deal with difficult situations and the stress involved in looking after people with haemophilia.

HPs from other centres explained that patients have to be referred to psychosocial services that are provided by a separate team within the hospital, an associated hospital or in the community. They felt frustrated and concerned for patients who often struggled to access support services.

You would try and refer for psychological support but we don't actually have anything within the unit and we don't have anything to access. We’ve had a real difficulty with a patient who really does need some long-term therapy, and we don't have that. We have it for patients with HIV or hepatitis and trying to get that in the community is very difficult and very challenging, they don't have the resources and it's so frustrating ‘cos you can see that he needs it and he wants it (HP4)

Most of the HPs felt that a multidisciplinary team that has access to play a therapist and social worker, but includes a psychologist on a permanent basis is the ideal set-up. This would mean that patients would see the psychologist as part of the team and the threshold to access support would, therefore, be low. Rather than patients having to see a psychologist separately once potential issues arise, an in-house psychologist may be able to identify issues early and support patients and their families through challenging periods without the need for significant psychological interventions.

Whereas I think if we had access to someone most of the time or a lot of the time so this person's not a complete alien to these patients […] I can't help but think that some of the problems that you do see these patients going through could be nipped in the bud. You wouldn't get so bad that you’re having to refer them to a counsellor, it's something that might have been able to have been dealt with before it became a big problem. (HP2)

In addition to psychological support, HPs felt that peer support groups or activities can be very beneficial. Some haemophilia centres facilitate meetings between young patients to provide social support as well as facilitating patients to learn from each other. Two HPs explained that they occasionally bring together non-adherent patients with adherent patients to try and encourage non-adherent patients to get back on track with their treatment.

I think it went very well because the one who didn't treat was able to see someone of the same age, and see how he managed to do it himself. And he showed him – and the other boy who didn't treat himself was able to actually do it himself and it's given him confidence because he didn't seem like the most – one child was quite a confident child and the other one was not such a confident personality and I think it was good for them and I believe they even exchanged numbers. It just gives them a chance to meet someone else with the same condition. (HP3)

However, HPs appeared frustrated that the current pressures on budgets and resources have made it harder and sometimes impossible to organise regular groups or activities locally.

In the context of the more flexible approach to prophylaxis, HPs emphasised that personalising care is not just about treatment, but also about the way you approach a patient and communicate with them. HPs try and tailor their approach to individual patients as much as possible by, for instance, providing telephone appointments to accommodate people who work long hours or contacting patients in their preferred way (e.g. email, text or telephone), but also by being mindful of any personal issues the patient has and the tone and language they use. Although prophylaxis is the treatment of choice, HPs appreciate that for some patients it is simply not possible to follow a prophylactic regimen. They often try a range of different strategies that take the individual patient's circumstances into account, but sometimes have to accept that a patient does not want to take prophylaxis. In those cases, they shift their focus to making sure that the patient does not disengage completely, and sometimes this means that a patient is moved to an on-demand regimen to treat bleeds if and when they occur. In other cases, they may reach a compromise with a patient or family that reduces the treatment burden while still providing some prophylactic cover.

So although there's lots of positives of daily treatment and that would be ideal in terms of levels. The practicality of that for some families is that it's not going to work. I suppose the compromise then is alternate days or we say “Monday, Wednesday and Friday and once at the weekend’’. If that's what's prescribed, I would say 90–100% of people are doing that. I would say particularly in children that less than 50% of them would be doing it in the morning. I think it's after school, in the evening. (HP6)

HPs appear passionate about supporting their patients and believe that a good relationship between patients and the haemophilia team can be crucial in keeping patients on track. HPs felt that maintaining a good relationship with patients used to be more straightforward, as patients had to come to the haemophilia centre frequently to receive treatment, or to pick up their home treatment. These visits offered an opportunity to catch patients for an informal chat and enabled the team to identify potential issues quickly. Now that many patients get their treatment delivered to their home, contact has become less frequent, with some patients only coming to the haemophilia centre once or twice a year. HPs felt that as a result, some patients may become less engaged with the haemophilia team and their treatment, potentially leading to non-adherence. They explained that when contact with a patient breaks down (i.e. when they do not attend clinic appointments or do not answer telephone calls or letters from the haemophilia centre) it can be an indication that the patient is not keeping to their treatment regimen and not treating bleeds appropriately. It can be very challenging and sometimes frustrating to try and re-engage these patients. HPs explained that they tend to spend a lot of time and effort trying to contact and chase patients that they are concerned about.

They agreed that this tends to be a small group of patients who take up a disproportionate amount of time because they often do not respond to letters or telephone calls and require regular follow-ups. When this significant effort does not result in better adherence (resulting in more regular bleeds) this can be frustrating for HPs who care about their patients and are genuinely worried when a patient goes ‘off radar’. In particular, nurses felt that they support patients in many more ways than specified in their job description. They appear to go above and beyond to help and support patients. HPs suggested that staff turnover is low in haemophilia centres, and that as a result the haemophilia team and patients get to know each other well, and often develop a close relationship. Visiting families at home can help with establishing a good relationship, and identifying potential issues that would be difficult to ascertain in clinic.

But I think once you start actually going and seeing them outside the hospital it gives you a completely different dynamic on things and them you, and sometimes it becomes a lot more personal and you are more involved in their lives, you’re more involved because you’re watching them grow-up and they know you, which is nice and it helps to some extent because they’ve got a better trust and a better bond with you. (HP2)

Although the close relationships between the haemophilia team and their patients was generally seen as positive, it was also recognised that it can lead to over-dependency on a specific nurse or doctor. One nurse explained that she regularly gets contacted on her personal mobile phone by patients, another joked that nurses do not need to say their name when they answer the phone as patients immediately recognise their voices.

In addition to working with patients and their families directly, haemophilia teams try to engage and collaborate with schools and community teams as much as possible. Schools are an important priority, as young patients spend a significant amount of time at school and may take part in potentially risky activities, such as PE lessons. HPs explained that they also try to educate schools about what haemophilia is, and what it means for the patient and their family, to encourage schools to focus on supporting the patient rather than just managing the risks.

## Discussion

### Findings

HPs felt that adherence among YPH is generally good, although they explained that levels of adherence are likely to fluctuate, with even very adherent patients experiencing short periods of non-adherence. As prophylactic regimen are increasingly personalised and flexible, it is much harder to ascertain if a patient is non-adherent. In addition to the existing designations of non-adherence due to forgetting and skipping, with a personalised regimen it is also possible to be non-adherent accidentally. In many cases, haemophilia teams only intervene if a patient is presenting with bleeds or not attending check-up appointments, as they are often an indication that someone is not engaged with their treatment, and may be struggling with self-management.

Time management and lifestyle-related issues were suggested to be key reasons why patients do not adhere to their treatment. During a busy time, or significant life event or change, someone is less likely to adhere. Most HPs agreed with the existing literature (e.g. Breakey, Blanchette, & Bolton-Maggs, 2010; Schrijvers, Schuurmans, & Fischer, 2016; Thornburg, 2008) that adherence may be a particular issue during adolescence, as a result of the specific developmental issues that characterise this period. It can be challenging for haemophilia teams to engage with adolescent patients, particularly if they rebel against the haemophilia team and parents. Other reasons for non-adherence that were mentioned were issues related to venous access; absence of symptoms; family dynamics (relationship with and support from parents and siblings); psychosocial issues; and a lack of knowledge about haemophilia and treatment.

HPs felt that a multidisciplinary team that, ideally, includes a psychologist and/or social worker would be the ideal set-up to support patients (and families) who are struggling with prophylaxis. However, in most haemophilia centres psychosocial support is only available through referral to a psychologist, which is often difficult due to a lack of funding and resources. Participants also felt that bringing YPH together through peer support groups or activities is beneficial, but that the current pressures on budgets and resources have made it harder and sometimes impossible to organise support groups or activities locally.

It is clear that HPs are passionate about supporting patients in as many ways as they can, and motivated to keep patients on track with their treatment to ensure optimal health outcomes. HPs believe that a good relationship between patients and the haemophilia team can be crucial in keeping patients on track. They tend to spend a lot of time and effort trying to contact and chase patients that they are concerned about, and agreed that this tends to be a small group of patients who take up a disproportionate amount of time. Their work is made challenging by pressures on resources, patients’ social and psychological issues, and limited understanding of haemophilia within and outside of the medical profession. However, it appears that they manage to maintain their motivation to do their best, which is very clearly appreciated by patients (van Os et al., 2018).

### Strengths and limitations

The strength of this qualitative study is that it recruited from four centres across England and Wales. The findings represent views of a range of HPs, who are likely to have a range of experiences and perceptions in relation to haemophilia and prophylaxis.

The most important limitation is that the interview data represent the lived experience of individual participants, which are not necessarily representative of the whole population. It is also impossible to know whether the lived experiences represented in interview data remain unchanged over time as individual circumstances, experiences and perceptions may fluctuate or change.

### Implications

Notwithstanding the limitation discussed above, the findings of this study have a number of implications. The findings support previous quantitative evidence that adherence is generally good amongst YPH (van Os et al., 2018). However, the findings on the experience of HPs in the present study also complement the experience of patients reported in van Os et al. (2018). For example, while patients identified barriers and facilitators of adherence (such as anxiety and inconvenience versus treatment being a routine), HPs identified broader drivers of non-adherence such as absence of symptoms and family/social issues. In addition, while patients identified the importance of support from family, friends and haemophilia centres, HPs identified factors that would improve adherence more broadly, including organisational factors (psychological services, continuity of haemophilia staff), community factors (e.g. involvement of schools), peer support and HPs being sensitive to individual needs. Furthermore, while patients identified the tension between good self-management and living the life they want, HPs identified, for example, the fluctuating nature of adherence and the role of treatment logs to instigate a conversation about adherence.

Haemophilia teams tend to have good relationships with many of their patients. These relationships are often maintained by regular contact, particularly with those patients who are struggling. In the current economic climate, and in the context of reorganisation and rationalisation of the NHS, this model of care may come under increased pressure and may not be able continue if resources are cut. However, the findings from this study provide evidence for the benefits of the current approach in managing patients with severe haemophilia, and may, therefore, help haemophilia centres to build a case to retain their current level of resource.

Many patients require, and indeed access, additional support to help them with the psychological and social impact of haemophilia. All HPs agreed that psychological support is an important element of the comprehensive care that many haemophilia patients require. They felt that this support should ideally be provided within the haemophilia centre setting so that the threshold to access this support is low and psychologists are able to work with patients pro-actively to address issues before they escalate. HPs felt strongly that regular psychological input would contribute towards better patient outcomes, particularly for patients who struggle or are disengaged from their treatment. However, several of the centres involved in this research no longer have (or never had) a psychologist embedded in their team. The findings of this research may help centres to put forward an argument to improve access to psychological support, or indeed appoint a psychologist in their centre.

The findings also indicate that, due to the increasingly flexible approach to haemophilia treatment in the U.K., adherence to prophylaxis is difficult to define and assess. HPs agreed that they often prioritise working with patients who are clearly struggling, as indicated by bleeding episodes or other haemophilia-related issues they present with. This is partial because they have to prioritise due to limited resources and time, but also because patients who are not bleeding are often assumed to be doing well, even if they are not adhering to their treatment or feel unable to pursue particular activities for fear of bleeding.

The findings of this research suggest that it may be useful to shift the focus of future research away from looking for ways to improve adherence generally, but rather to focus on improving adherence among those patients who are likely to have worse outcomes due to sub-optimal adherence. It would be useful for the haemophilia healthcare community to discuss adherence in this wider perspective, to come to a nationwide agreement of what good adherence looks like in the context of improving outcomes in this patients group.

## Conclusion

The increasingly flexible approach to prophylaxis requires a new way of thinking about, and assessment of, adherence. More personalised treatment plans can be more complicated and can, therefore, lead to confusion and result in accidental non-adherence. Some additional training and education of patients and their families are likely to reduce this confusion, and therefore prevent accidental non-adherence. HPs felt quite strongly that patients would benefit from ongoing routine psychological support, ideally provided by a psychologist who is a member of the haemophilia team. This would allow the team to identify potential issues earlier and address these before they escalate, and would make it much easier for patients to access support when they need it.
